# Metabolic engineering of raffinose-family oligosaccharides in the phloem reveals alterations in carbon partitioning and enhances resistance to green peach aphid

**DOI:** 10.3389/fpls.2013.00263

**Published:** 2013-07-19

**Authors:** Te Cao, Ipsita Lahiri, Vijay Singh, Joe Louis, Jyoti Shah, Brian G. Ayre

**Affiliations:** Department of Biological Sciences, University of North TexasDenton, TX, USA

**Keywords:** raffinose family oligosaccharides, green peach aphid, phloem transport, sugar transport, metabolic engineering

## Abstract

Many plants employ energized loading strategies to accumulate osmotically-active solutes into the phloem of source organs to accentuate the hydrostatic pressure gradients that drive the flow of water, nutrients and signals from source to sinks. Proton-coupled symport of sugars from the apoplasm into the phloem symplasm is the best studied phloem-loading mechanism. As an alternative, numerous species use a polymer trapping mechanism to load through symplasm: sucrose enters the phloem through specialized plasmodesmata and is converted to raffinose-family oligosaccharides (RFOs) which accumulate because of their larger size. In this study, metabolic engineering was used to generate RFOs at the inception of the translocation stream of *Arabidopsis thaliana*, which loads from the apoplasm and transports predominantly sucrose, and the fate of the sugars throughout the plant determined. Three genes, *GALACTINOL SYNTHASE, RAFFINOSE SYNTHASE* and *STACHYOSE SYNTHASE*, were expressed from promoters specific to the companion cells of minor veins. Two transgenic lines homozygous for all three genes (GRS63 and GRS47) were selected for further analysis. Three-week-old plants of both lines had RFO levels approaching 50% of total soluble sugar. RFOs were also identified in exudates from excised leaves of transgenic plants whereas levels were negligible in exudates from wild type (WT) leaves. Differences in starch accumulation between WT and GRS63 and GRS47 lines were not observed. Similarly, there were no differences in vegetative growth between WT and engineered plants, but the latter flowered slightly earlier. Finally, since the sugar composition of the translocation stream appeared altered, we tested for an impact on green peach aphid (*Myzus persicae* Sulzer) feeding. When given a choice between WT and transgenic plants, green peach aphids preferred settling on the WT plants. Furthermore, green peach aphid fecundity was lower on the transgenic plants compared to the WT plants. When added to an artificial diet, RFOs did not have a negative effect on aphid fecundity, suggesting that although aphid resistance in the transgenic plants is enhanced, it is not due to direct toxicity of RFO toward the insect.

## Introduction

Phloem loading is the energized accumulation of solutes, principally sugars, from mesophyll cells into the sieve element/companion cell complex (SECCC) of the phloem. This accumulation generates hydrostatic pressure that contributes to bulk flow of phloem sap from source to sink tissues. Two mechanisms, apoplasmic loading and symplasmic loading, are well-characterized (Turgeon and Ayre, 2005). In the most prevalent form of apoplasmic phloem loading, sucrose (Suc) from the mesophyll is released into the cell wall space (i.e., the apoplasm) via SWEET transporters (Chen et al., 2012) and is loaded across the plasma membranes of the SECCC by Suc/proton (H^+^) symporters (Suc uptake transporters, SUT, or Suc uptake carriers, SUC). The energy for SUT-mediated phloem loading is provided by the proton motive force that exists between the apoplasm and the SECCC symplasm (Ayre, 2011). Symplasmic phloem loading operates by a polymer trapping mechanism, in which Suc diffuses into intermediary cells (specialized companion cells) from mesophyll cells through narrow and highly branched plasmodesmata. Inside intermediary cells, Suc is polymerized to raffinose family oligosaccharides (RFOs) which are thought to be too large to diffuse back across the specialized plasmodesmata (Turgeon and Gowan, 1990; Turgeon, 1996). In this strategy, conversion of Suc to RFOs maintains a favorable Suc concentration for diffusion while RFO accumulation provides osmotic potential to drive long-distance transport. More recently, it was shown that some species have the highest solute concentration in mesophyll cells and plasmodesmata connections that permit passive flux of sugars into the phloem translocation stream (Turgeon and Medville, 1998; Rennie and Turgeon, 2009). Although this is commonly called passive “loading,” there is no energized concentrating step and these species technically do not engage in phloem loading.

The objectives of the studies reported here were to assess the impact of engineering symplasmic phloem-loading biochemistry into a plant that normally loads Suc from the apoplasm with SUTs, and follow the fate of the resulting RFO sugars. *Arabidopsis thaliana* uses the SUT encoded by *AtSUC2* to phloem load Suc from the apoplasm (Truernit and Sauer, 1995; Gottwald et al., 2000). For the experiments reported in this manuscript, metabolic engineering was used to produce the RFOs raffinose (Raf; α D-Gal-[1-6]-α D-Glc-[1-2]-β D-Fru) and stachyose (Sta; α D-Gal-[1-6]-α D-Gal-[1-6]-α D-Glc-[1-2]-β D-Fru), along with the RFO precursor galactinol (Gol; α D-Gal-[1-3]-1D-myo-inositol), specifically in the Arabidopsis minor-vein companion cells: i.e., RFOs were synthesized after the SUT-mediated phloem loading step. The aims of these experiments were 2-fold: (1) to assess the efficiency of RFO synthesis in the phloem and (2) to gauge the efficiency with which RFO from the companion cells enters the translocation stream for long-distance transport. Arabidopsis is well-adapted to synthesize RFOs since, like many other species, it will induce RFO synthesis in response to cold, drought, heat and other stresses, presumably to serve as compatible solutes (Liu et al., 1998; Taji et al., 2002; Nishizawa et al., 2008). Low levels are detected in phloem sap, but Arabidopsis does not use these as prominent transport sugars (Haritatos et al., 2000b). Three genes encoding the biosynthetic enzymes galactinol synthase (GolS; Inositol 1-α-galactosyltransferase; EC 2.4.1.123), raffinose synthase (RafS; Gol-Suc galactosyltransferase; EC 2.4.1.82) and stachyose synthase (StaS; Gol-Raf galactosyltransferase; EC 2.4.1.67) were expressed from minor vein and companion cell specific promoters: *GALACTINOL SYNTHASE 1* (*CmGAS1)* from melon (*Cucumis melo*) and the *RAFFINOSE SYNTHASE* (*CsRFS*) from cucumber (*Cucumis sativus*) were expressed from the *CmGAS1* promoter, which confers gene expression to the minor veins (Haritatos et al., 2000a; Ayre et al., 2003a) and *STACHYSOSE SYNTHASE* (*AmSTS1*) from Alonsoa (*Alonsoa meridionalis*) was expressed from the *Mature Minor Vein Element1* (*MMVE1*) (McGarry and Ayre, 2008).

We report on the accumulation and transport of RFOs in the transgenic plants, and their effect on growth and development. We also report on the impact of these manipulations on the behavior of a prevalent phloem-feeding insect, the green peach aphid (*Myzus persicae* Sulzer). The potential of converting natural phloem sugars to exotic transport molecules after the phloem loading step is discussed in a broad context.

## Materials and methods

### Plasmid constructions

pGPTV-Hyg-CmGAS1p:CmGAS1 was previously described (Ayre et al., 2003b) and consists of a 5 kb genomic sequence from *Cucumis melo* (muskmelon) subcloned in the *EcoRI* site of pCambia1301. pGPTV-Kan-*CmGAS1p:CsRFS* contains a phloem-specific *RAFFINOSE SYNTHASE* (*CsRFS*; NCBI accession no. AF073744) cDNA from *Cucumis sativum* (cucumber) downstream of the *CmGAS1* promoter. RNA from muskmelon seedlings was isolated with Trizol (Life Technologies, Carlsbad, CA) and reverse transcribed with Superscript RT II (Life Technologies) and an oligo dT primer according to the manufactures instructions. *CmRFS* cDNA was PCR-amplified with Vent DNA polymerase (New England Biolabs, Ipswich, MA) and oligonucleotides Raf5Kpn 5′-TCTTCTGGTACCAACAATGGCTCCTAGTTTTAAAAATGGTGG-3′ and Raf3Xma 5′-TACATAGAGCTCAACCCCGGGAAAACAAGTACTCGATAACCGAA-3′. The PCR product was phosphorylated with polynucleotide kinase (NEB) and circularized by ligation with T4 DNA ligase (NEB). An internal SacI site was removed with Vent polymerase and mutagenic oligonucleotide pairs RafSmut 5′-CAGTGATGAGTTCAAATTCGAATGG-3′ and RafSmut2 5′-CCATTCGAATTTGAACTCATCACTG-3′. The mutagenized cDNA was created by a final PCR with oligonucleotides Raf5Kpn and Raf3Xma, digested with KpnI and SacI restriction enzymes (NEB) and inserted into the same sites of pUC-GUT:T7CO (Ayre and Turgeon, 2004). The *CmGAS1* promoter:*CsRFS* cDNA cassette was inserted into pGPTV-Kan (Becker et al., 1992) as a SbfI—SacI cassette.

pGPTV-Bar-*IAA11p:AmSTS* contains *STACHYOSE SYNTHASE* from *Alonsoa meridionalis* (*AmSTS1*; NCBI Genbank accession no. AJ487030) downstream of the minor-vein and companion-cell-specific *MMVE1* promoter element (McGarry and Ayre, 2008). Alonsoa loads through the symplasm by polymer trapping and predominantly translocates stachyose. Alonsoa mRNA was isolated and reverse transcribed as described above; *AmSTS1* cDNA was PCR amplified with oligonucleotides StaSKpnF 5′-ATTAGAGGTACCATGGCACCTCCATATGATCC-3′ and StaS2604R 5′-GAGCTCAAGCCCGGGGGTAAACAAAAGTTACATTAGAAATCC-3′. Mutagenic PCR was used as described above to remove internal SacI and XmaI sites using oligonucleotide pairs StaS78F 5′-GACAACTCTTTCGAACTCCTCGACG-3′ and StaS78R 5′-CTTTACGGTGCTGCTCAGGAAGTTC-3′, and StaS1825 5′-GAGGATACTTTCACGCGGGATCAA-3′ and StaS1825R 5′-CCGGGTGATCGGATTGAAACATGTC-3′. The mutagenized *AmSTS1* cDNA was created with a final PCR with StaSKpnF and StaS2604R, digested with KpnI and SacI and inserted into the same sites of pUC-IAA11p:T7CO (McGarry and Ayre, 2008) and the *IAA11p*:*AmSTS1* cassette was inserted into pGPTV-bar (Becker et al., 1992) with SbfI and SacI. All sequences generated by PCR were sequenced for accuracy (SeqWright Inc., Houston, TX). Each engineered binary vector was electroporated into *Agrobacterium tumefaciens* GV3101 mp90 (Koncz and Schell, 1986).

### Plant materials

*Agrobacterium* cultures containing the T-DNA plasmids were grown overnight, and equal amounts of each, based on optical density at 600 nm, were mixed for simultaneous floral dip transformation (Clough and Bent, 1998) of Arabidopsis Col-0. T1 seedlings harboring T-DNA from each construct were selected on sterile Murashige-Skoog (MS) medium with Gamborg vitamins (PhytoTechnology Laboratories, Shawnee Mission, KS) supplemented with 1% Suc, hygromycin B (40 mg/L), kanamycin sulfate (100 mg/L), glufosinate ammonium (10 mg/L; all from PhytoTechnology Laboratories), pH 5.8 with KOH, and solidified with Gel-Rite gellan gum (2.8 g/L; Sigma). Seeds were gas sterilized by acidifying chlorine bleach in a sealed desiccator jar (Clough and Bent, 1998) and the method of Harrison and colleagues (Harrison et al., 2006) was used for selecting transgenic seedlings as it was particularly well-suited for identifying hygromycin resistance. Two independent lines, GRS47 and GRS63 were carried through several generations until the segregation patterns indicated they were homozygous for all three T-DNA inserts. Subsequently, all plants were grown on potting mix (Metromix 360, Scotts Horticultural) without selection in Percival growth chambers (Percival Scientific, Inc. Perry, IA) with 110–150 μmol photons m^−2^ s^−1^ and 14/10 h light/dark cycles. For analysis of vegetative growth, transgenic and wild-type (WT) plants were photographed and the total rosette area calculated using ImageJ software (Rasband, 2007). Plants were also monitored for days to flowering, and the number of rosette leaves at flowering.

### Carbohydrate analysis

Carbohydrate levels in rosettes of GRS47, GRS63, and WT controls were analyzed 21 days after germination (dag) at the end of the dark period and 8 h into the light period. Sugar extraction with methanol:chloroform:water (MCW) and quantification of soluble sugars by high-performance anion exchange chromatography (HPAEC; Dionex, Sunnyvale, CA) was as previously described (Srivastava et al., 2008), using 10 μM lactose as a recovery standard instead of 100 μM. Elution conditions were optimized for separation of the six major sugars, Glc, Fru, Suc, Gol, Raf, and Sta. Under these conditions, Glc and Gal elute together, but in separate elutions, levels of Gal were always negligible. Starch was quantified with the Total Starch (AA/AMG) kit, scaled down 100× (Megazyme, Bray, Ireland). For micro-dissection of areoles (leaf sections bounded by, but not including vein tissue) and phloem-enriched tissues, mature leaves that had recently achieved full expansion were cut at the petiole and quickly but carefully placed between two sheets of 3MM paper (Whatman) and flash frozen in powdered dry ice. They were then lyophilized in a cooled chamber held at −20°C for 48 h, and subsequently stored in desiccant until processed. The sharp edge of a 22 gauge syrinige needle was used for dissection, and sugar extraction and analysis was is described above.

Phloem exudations from cut petioles were collected into EDTA solutions by modification of standard techniques (King and Zeevaart, 1974; Srivastava et al., 2008; van Bel and Hess, 2008). In brief, leaves 6 through 11 from plants 30 dag were excised at the stem and fresh weights were recorded. Petioles were cut again under 10 mM EDTA and arranged into a small chamber (Coulter Counter, Pittsburgh, USA) containing 2 ml of 10 mM EDTA, such that only the cut ends and less than 2 mm of petiole were submerged. The chambers were capped to maintain humidity and minimize the amount of solution drawn into the leaves by transpiration. Exudates from the first 20 min were discarded because there may be contamination from cut cells and subsequent exudates from each of two 2-h periods were collected, immediately frozen, and subsequently analyzed by HPAEC as described above. Consistency between values obtained from the first full 2 h of exudation and the second 2 h of exudation were consider indicative of minimal contamination of exudates with solutes and/or enzymes from damaged cells.

Since the promoters used are specific to the minor veins of leaves, the sugar content of roots was analyzed as an additional indicator of long-distance transport. GRS47, GRS63 and WT seeds were sterilized, stratified and germinated on MS medium with 1% Suc but without antibiotics. After 7 d, seedlings were transferred to fresh MS medium without Suc and with 4.8 g/L gellan gum in square plates, such that the rosettes were in a line at the top of the plate and the roots trailed behind on the medium surface; the plates were sealed with parafilm and oriented in the growth chamber 15° from vertical for an additional 9 and 14 d (16 and 21 d total). Rosettes and roots were collected separately and analyzed for soluble carbohydrates by HPAEC as described above.

For photosynthetic labeling, seeds were sterilized, stratified and germinated on MS medium with 1% Suc and transferred to MS medium without Suc for growth in vertically-oriented plates as described above. After 7 d growth in the absence of supplemental Suc, the plates were placed horizontally 60 cm below a 400 W metal halide lamp and the lid of each replaced with another lid lined with vacuum grease for a tight seal, and having two small holes at the top and bottom. ^14^CO_2_ was generated and injected into the plates by mixing 5 μ L of 1 μ Ci/mL NaH^14^CO_3_ (MP Biochemicals) with 15 μ L of lactic acid in the barrel of a 1 mL syringe with a 22 gauge needle extending through the hole in the top hole of the plate, avoiding injection of the lactic acid solution. After 20 min, residual ^14^CO_2_ was withdrawn through the hole at the bottom of the plate and passed over soda lime by a small aquarium-style air pump for 5 min; plants were left to photosynthesize in ambient air for 10 min and sugars were extracted as described above.

Radioactive sugars were resolved by one dimensional thin-layer chromatography essentially as describe (Turgeon and Gowan, 1992). In brief, silica gel TLC plates (Analtech, Newark, NJ) were pre-treated by dipping in 0.03% boric acid in 60% ethanol and activated at 110°C for 1 h. Four μ g of unlabeled sugar standards and extracts corresponding to 25 mg fwt were spotted, dried, and developed in closed TLC tanks with a solvent composed of 60 mL chloroform, 70 mL acetic acid and 10 mL water. Once the solvent front reached the top of the plate (~2 h), the plate was allowed to air dry and then immediately subjected to a second development to further resolve the spots. Gol and Raf co-migrate under these conditions. To visualize the spots, the plates were sprayed with a vanillin solution (3 g vanillin and 0.5 mL concentrated sulfuric acid in 100 mL absolute ethanol) and heated at 100°C for 20–30 min. Autoradiography was with Kodak BioMax film, developer, and fixer, according to the manufacturer's instructions. Identification of labeled sugars was by co-migration with standards, and relative levels of isotope in each were quantified by radiometric scanning (Bioscan System 2000 Image Scanner, Bioscan, Washington, DC).

### Gene expression analysis

Total RNA was extracted from two biological replicates, each consisting of leaves pooled from two plants, 22 dag: Leaves were ground in liquid nitrogen and extracted with Trizol using the manufacturer's protocol (Life Technologies). The isolated RNA was treated with DNase, spectrophotometrically quantified, and cDNA synthesis was performed with Superscript III Reverse Transcriptase (Life Technologies) using an oligo dT primer, as per the manufacturer's protocol. RT-qPCR was with the Sybr Green PCR Master Mix (Life Technologies) according to the manufacturer's instructions. The oligonucleotides for *CmGAS1* mRNA were GolRTbFwd 5′-AGCCCATTCCTCCCATTTAC-3′ and GolRTb Rev 5′-TCTCTTCTTTTCCCGTGTAC-3′; for *CsRFS*, RafRTbFwd 5′-GGTGTGGTGAGATGCGAGTA-3′ and RafRTbRev 5′-CCGAAATGCCACCCGATGAA-3′; for *AmSTS*, StsRTaFwd 5′-CCATTTTGCTCTCCCGACTA-3′ and StsRTaRev 5′-CTTGATCCTTTGCTCCTTCG-3′; and for the *EF1a* internal standard, EF1α Fwd 5′-GAGACTCGTGGTGCATCTCA-3′ and EF1α Rev 5′-AGGTCCACCAACCTTGACTG-3′. The RT-qPCR was performed on a Eco qPCR system (Illumina, San Diego, CA) with denaturation at 95°C for 5 min followed by 40 cycles of 95°C for 10 s, annealing at 60°C for 30 s, and extension at 72°C for 35 s.

### Aphid feeding experiments

“No-choice” experiments were conducted to test aphid fecundity on GRS47, GRS63 and WT plants 30 dag (Pegadaraju et al., 2005). Twenty adult aphids were put on the rosette of each plant and after 2 days the number of aphids (adults plus nymphs) on each counted. “Choice” experiments were conducted to establish if a feeding preference between WT and RFO over-producing plants existed (Louis et al., 2010). Twenty adult aphids were placed on the soil equidistant between a WT and a transgenic plant growing in the same 15 cm diameter pot and the number of adult aphids on each was counted after 8 and 24 h. To test if Glc, Fru, Suc, Gol, Raf or Sta directly influenced aphid vitality, an artificial aphid diet (Mittler and Dadd, 1965) was supplemented with 50 mM of each sugar and tested in a feeding chamber: Three adult aphids were transferred with a fine paintbrush into a 3.5 cm Petri dish (Falcon, Primaria, NJ) and pre-stretched parafilm (American National Can, Greenwich, CT) was placed over the chamber to cage the aphids. 500 μL of diet was placed on the parafilm and a second layer of pre-stretched parafilm was placed on the first layer to spread the diet and form a feeding sachet from which the aphids could feed. Aphid numbers (adults and nymphs) were counted 4 days later (Louis et al., 2010).

## Results

### Carbohydrate steady state and flux analysis in mature leaves

**Figure 1:**
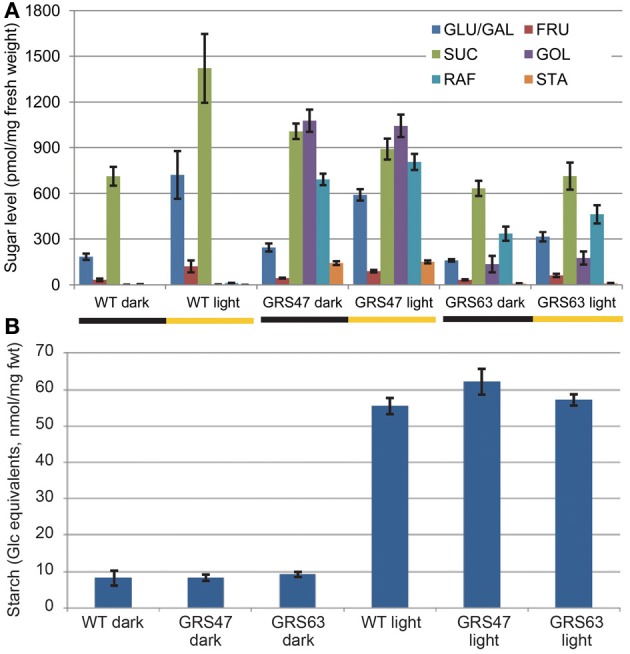
**Comparison of carbohydrate levels after 10 h of dark and 8 h into the 14 h illuminated period. (A)** Soluble neutral sugars as indicated (pmol sugar/mg fresh weight) (note: separation conditions were optimized for Gol, Raf, and Sta, and Glc and Gal elute together. In elutions that do separate Glc and Gal, Gal levels were always negligible). **(B)** Starch levels from the same samples as **(A)**, expressed as Glc equivalents. Variation is expressed as SE; *n* = 6 sibling plants. Student's *t*-Test analysis was also conducted to assess differences (*p* < 0.05) in the level of each sugar within each line between 10 h of dark and 8 h of illumination. Glc, Fru, and starch were significantly increased in all lines, Suc increased significantly only in WT, and none of the RFO sugars in any line showed a significant difference between 10 h of dark and 8 h or illumination.

WT and transgenic lines GRS47 and GRS63 homozygous for *CmGAS1* (encoding galactinol synthase from melon, GolS), *CsRFS* (encoding raffinose synthase from cucumber, RafS) and *AmSTS* (encoding stachyose synthase from *Alonsoa meridionalis*, StaS) were analyzed for soluble sugars and starch in rosette leaves 21 dag at two time points: at the end of the 10 h dark period and 8 h into the 14 h illuminated period (Figure 1A). WT plants had negligible Gol, Raf and Sta, consistent with expectations for unstressed conditions. Transgenic line GRS47 accumulated substantial Gol and Raf, and lesser, but still highly significant levels of Sta. The combined levels of engineered sugars in this line exceeded the levels of the canonical sugars Glc, Fru and Suc (~1900 pmol/mg fwt total RFO compared to ~1300 pmol/mg fwt Glc, Fru plus Suc at the end of the dark period, and ~2000 pmol/mg fwt total RFO compared to ~1550 pmol/mg fwt Glc, Fru plus Suc 8 h into the illuminated period). GRS63 accumulated levels intermediate between WT and GRS47 levels. Gol, Raf and Sta levels showed no significant changes between the end of the dark period and 8 h into the light period. Suc, Glc and Fru increased at least 2-fold in WT plants after 8 h of photosynthesis, consistent with published results (Blasing et al., 2005; Lunn et al., 2006) while in the transgenic lines, Glc and Fru similarly increased, but Suc levels were the same. Starch levels were low at the end of the dark period and dramatically higher 8 h in to the light period, as expected, but there were no significant differences between WT and transgenic lines (Figure 1B).

**Figure 2:**
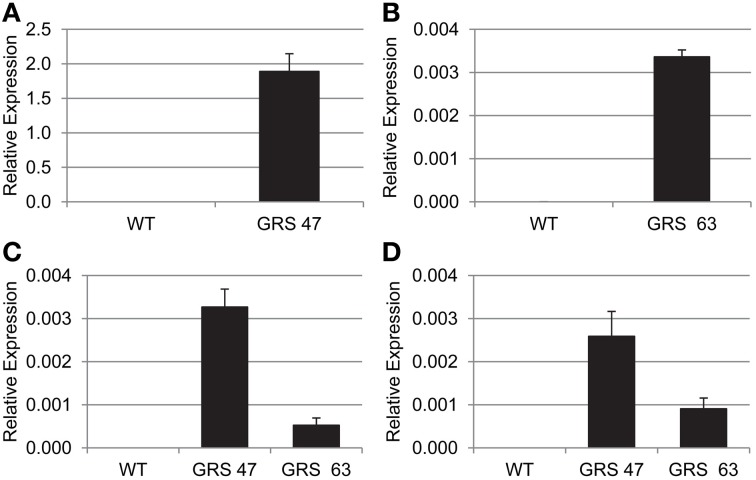
**Transgene transcript abundance as determined by RT-qPCR, relative to *EF1a* expression. (A)** *CmGAS1* expression in WT and GRS47 (note the Y-axis scale). **(B)** *CmGAS1* expression in WT and GRS63. **(C)** *CsRFS* expression in WT, GRS47 and GRS63. **(D)** *AmSTS* expression in WT, GRS47 and GRS63. Variation is expressed as SD; *n* = 4 sibling plants.

The accumulation of engineered sugar is consistent with transgene expression patterns determined by RT-qPCR (Figure 2): GRS47 expressed substantially more *CmGAS1* than GRS63 (>500x) and produced more Gol. Expression levels of *CsRFS* and *AmSTS* were also higher in GRS47 than GRS63, but not to the extent seen for *CsGAS*. The higher levels of Gol substrate and possibly higher enzyme activities in GRS47 are likely responsible for promoting the higher levels of Raf and Sta product.

Photosynthetic labeling with ^14^CO_2_ was conducted to gain insight into the flux of photosynthate through the engineered RFO pathway. After 20 min labeling, sugars were resolved by thin layer chromatography, and relative ^14^C incorporation into each sugar determined by autoradiography and radiometric scanning. For GRS47, in each of three replicates, less than 2% ^14^C was incorporated into Gol and Raf which co-migrate as a single spot, and a radio-labeled spot corresponding to Sta was not identified indicating very low incorporation. In contrast to these low levels, as much as 80% of the ^14^C was incorporated into Suc and as much as 30% was in spots corresponding to hexose sugars (results not shown). For both GRS63 and WT, labeled spots representing Gol, Raf, and Sta were not unambiguously observed, but label incorporation and distribution between Suc and hexose was similar to GRS47 (results not shown). Therefore, despite the high concentrations of RFO found in 21-d old plants, very little was created from photoassimilate during the labeling period. This argues that flux rate through the engineered pathway is very low, and that RFOs in rosettes are accumulating over a longer time.

RFO synthesis should be in the minor vein companion cells of GRS47 and GRS63 as a result of promoter specificity (Haritatos et al., 2000a; Ayre et al., 2003a; McGarry and Ayre, 2008) but are the RFOs confined to these sites of synthesis? In Arabidopsis, minor vein companion cells are generally 2–4 μm across and number 3–12 per minor vein (Haritatos et al., 2000b; McGarry and Ayre, 2008); vein density in Arabidopsis is low compared to other plants (2.45 ± 0.26 mm veins per mm^2^ of leaf area) (Haritatos et al., 2000b). Assuming a leaf is typically 200 μm thick and is 50% air space, the minor vein companion cells occupy ~0.0014 mm^3^ per mm^3^ of leaf tissue. If all RFO were resident in this space (~2000 pmol/mg fwt for GRS47), the concentration would be approximately 1.4 M. As indicated by plasmolysis experiments, solute concentrations in companion cells can exceed 1.4 OsM in several species, (Turgeon and Medville, 1998) but this includes the main transport sugars as well as all other solutes in the cells.

**Figure 3:**
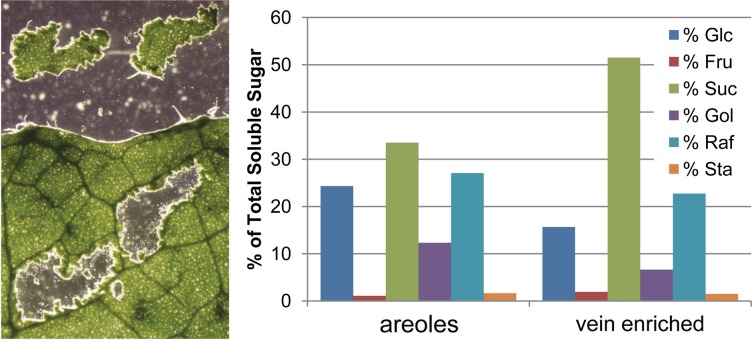
**Soluble sugars in areoles and vein-enriched sections. (Left)** Representative leaf that was flash frozen on powdered dry ice, lyophilized, and micro-dissected for areoles (sections containing only mesophyll cells and associated epidermis surrounded by minor veins). Vein-enriched sections (not shown) contained the minor veins and closely associated mesophyll that bordered the areole sections. **(Right)** Distribution of soluble sugars in pooled areoles and pooled vein-enriched sections, expressed as percentage of total detected sugar.

To determine if RFOs were confined to the phloem or distributed throughout the leaves, sections containing only areoles (areas of mesophyll surrounded by minor veins) and vein-enriched tissues surrounding the areoles were micro-dissected from flash frozen and lyophilized leaves, and analyzed for RFO content relative to total soluble sugar content (Figure 3). As expected, the vein-enriched sections contained more Suc and the areole sections had more Glc. As a percentage of total sugar, the areoles appear to have more RFO than the vein-enriched sections. This is somewhat misleading, however, since the high percentage of Suc in the veins drops the contribution of all other sugars. When Suc is not included in the total sugar calculation, RFOs are evenly distributed among the sections (not shown). From this, it is concluded that the engineered RFOs are evenly distributed throughout the leaves.

### Analysis of soluble sugars in phloem exudates and roots

**Figure 4:**
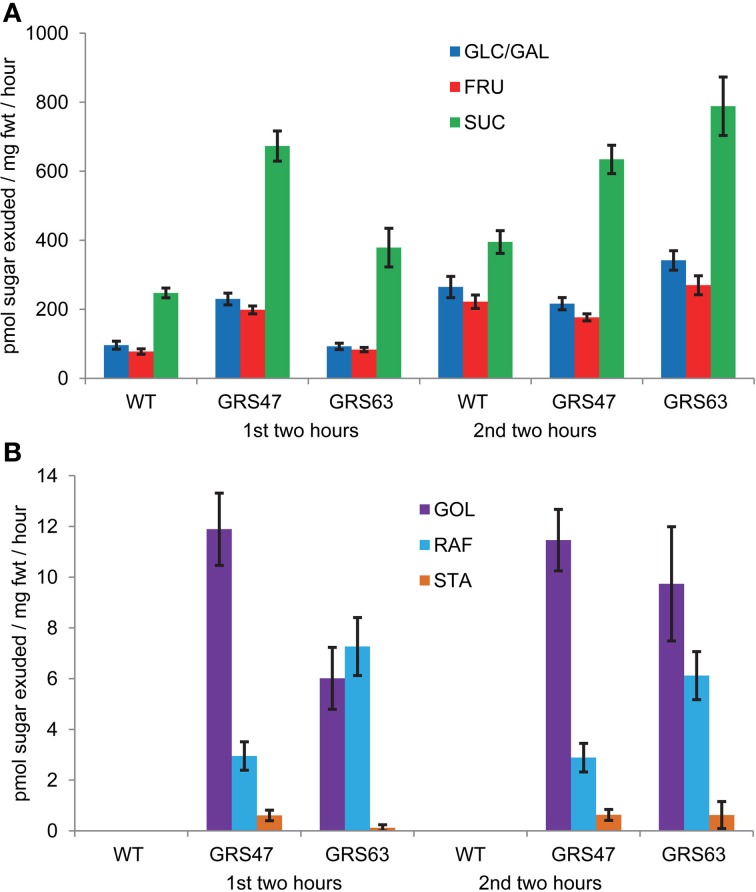
**Soluble carbohydrates in phloem exudates from wild type and transgenic plants. (A)** Exudation rates for Glc, Fru, and Suc. **(B)** Exudation rates for Gol, Raf, and Sta. Note the difference scales of the Y axis between **(A)** and **(B)**. Variation is expressed as SE; *n* = 12 sibling plants.

To investigate RFO transport efficiency, phloem exudates from cut petioles were collected into EDTA solutions for two consecutive 2-h periods and analyzed (Figure 4). As expected for Arabidopsis, Suc is the major transport sugar in both WT and transgenic plants. RFOs were negligible in exudates from WT but were present at low levels in the exudates of transgenic plants. This suggests that the engineered sugars are phloem mobile, but relative to Suc and other sugars, are insufficient to contribute substantially to phloem solute potential and pressure. Glc, Fru and especially Suc were present in the exudates of transgenic lines at greater levels than those of WT, suggesting that RFO degradation might be occurring. But, if the higher levels of Suc were the result of Raf and Sta degradation, then galactose levels would also be expected to be high. However, galactose levels were measured and were consistently negligible in all exudates, arguing that if degradation was occurring, it was not liberating Gal (not shown). Levels of Glc and Fru were relatively high (~25–50% of Suc) and roughly equal to each other in abundance, suggesting some invertase activity in the exudates (van Bel and Hess, 2008; Liu et al., 2012). Invertase would not impact Gol levels, but could convert Raf to Fru and melibiose (α D-Gal-[1-6]-α D-Glc) and Sta to Fru and α D-Gal-(1-6)-melibiose. These products were not measured.

**Figure A1:**
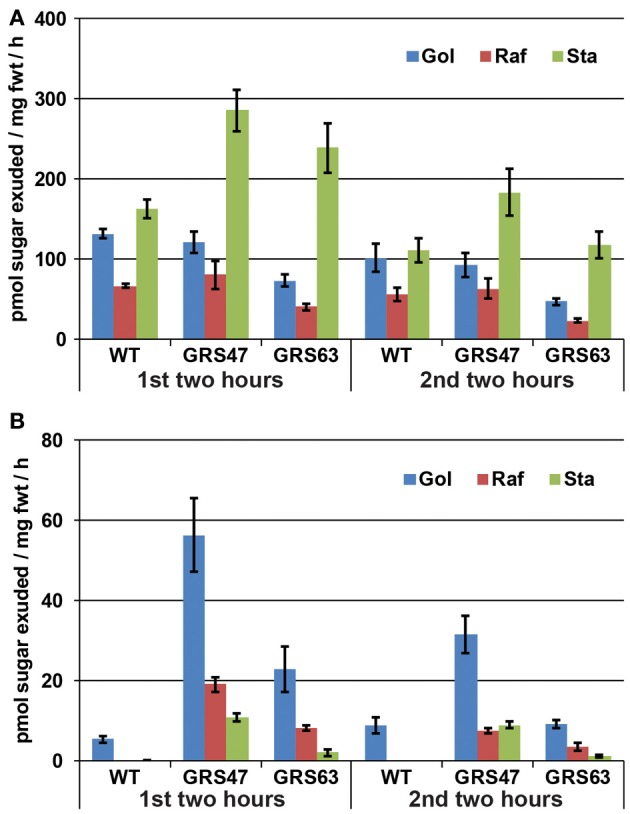
**Soluble carbohydrates in phloem exudates from wild type and transgenic plants**. This representative experiment is similar to that presented in Figure 4 except that they were conducted on 16 d-old whole rosettes with only the cut hypocotyl in EDTA to minimize exposure during exudations. In addition, EDTA was reduced to 5 mM. Similar to other exudation experiments, exudations were performed in dim conditions and 100% humidity to minimize EDFTA uptake through the xylem. **(A)** Exudation rates for Glc, Fru, and Suc. **(B)** Exudation rates for Gol, Raf, and Sta. Note the difference scales of the Y axis between **(A)** and **(B)**. Variation is expressed as SE; *n* = 6 sibling plants.

EDTA can damage cells and result in leakage of cellular contents (van Bel and Hess, 2008; Liu et al., 2012). Therefore, similar experiments were conducted on 16 d-old whole rosettes with only the cut hypocotyl in EDTA to minimize exposure during exudations. In addition, EDTA was reduced to 5 mM, which is cited as the minimum concentration that still prevents sealing of the sieve plates (van Bel and Hess, 2008), while still maintaining the plants in dim conditions and 100% humidity to minimize uptake through the xylem. The results of these experiments are shown in Figure A1, and are qualitatively similar to those shown in Figure 4, but overall the levels of Glc, Fru and Suc are lower, and RFO levels are higher among the transgenic lines. Differences in the age of the plants and use of whole rosettes rather than excised leaves may account for these changes, but the results are still consistent with long distance transport of RFO via the phloem. Despite these precautionary steps, a portion of the sugars detected may be from damaged cells.

Both exudation experiments suggest that GRS47, and to a lesser extent GRS63, are transporting more Glc, Fru, and Suc than WT, especially during the first full exudation period after the 20 min wash step (Figures 4, A1). The significance of this is not clear and this was not consistently observed in all exudation experiments (not shown).

**Figure 5:**
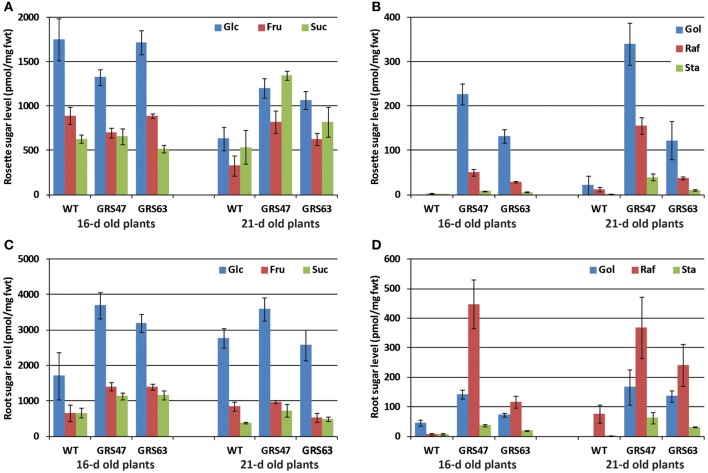
**Soluble carbohydrates in rosettes and roots of WT and GRS47 and GRS63 grown for 16 and 21 d on sterile MS media in vertically oriented plants. (A)** Glc, Fru, and Suc in the rosettes of each line at the indicated number of days, **(B)** Gol, Raf, and Sta in the same rosettes as indicated for **(A)**; note the difference in Y-axis scale. **(C)** Glc, Fru, and Suc in the roots of the same plants indicated in **(A)**, and **(D)** Gol, Raf, and Sta in the roots of the same plants indicated in **(A)**. All sugar values are averages expressed as pmol/mg fresh weight, *n* = 4, and variation is expressed as SE.

Since the promoters used in this study are specific to the minor veins of mature leaves (Haritatos et al., 2000a; McGarry and Ayre, 2008), plants were grown on MS medium in vertically oriented plates so that the heterotrophic roots could be readily accessed and analyzed for soluble sugars at 16 and 21 dag (Figure 5). Compared to older plants growing on potting mix, younger rosettes growing on plates had higher proportions of Glc and Fru relative to Suc (compare Figure 5A to Figure 1A). Gol, Raf, and Sta levels were lower than observed in the older plants on potting mix (compare Figure 5B to Figure 1A). Among WT, GRS47, and GRS63 plants growing on plates, the relative amounts of Glc, Fru, and Suc were similar but with modest differences in absolute levels, in both rosettes and roots (Figures 5A,C). The absolute levels of sugars varied between 16 and 21 dag, but the ratios of Glc, Fru, and Suc to each other were the same at each time among the three lines. In the rosettes of plants grown on plates, and consistent with results from plants grown on potting mix, GRS47 had more RFO than GRS63, and WT had very low to negligible levels (Figure 5B). Roots of WT plants on plates similarly had very low to negligible levels of RFO, as expected, but roots of GRS47 and GRS63 had substantial amounts (Figure 5D): Gol was between 50 and 70% of the levels in rosettes and Raf and Sta levels both exceeded those observed in rosettes. These results are consistent with long distance RFO transport via the phloem, and accumulation over time. Note that results in Figure 5 are relative to fresh weight and Figure 5C suggests RFO accumulation slightly faster than rosette growth, and Figure 5D suggests RFO accumulation in the roots at a rate roughly equal to the growth rate.

### Growth and development

**Figure 6:**
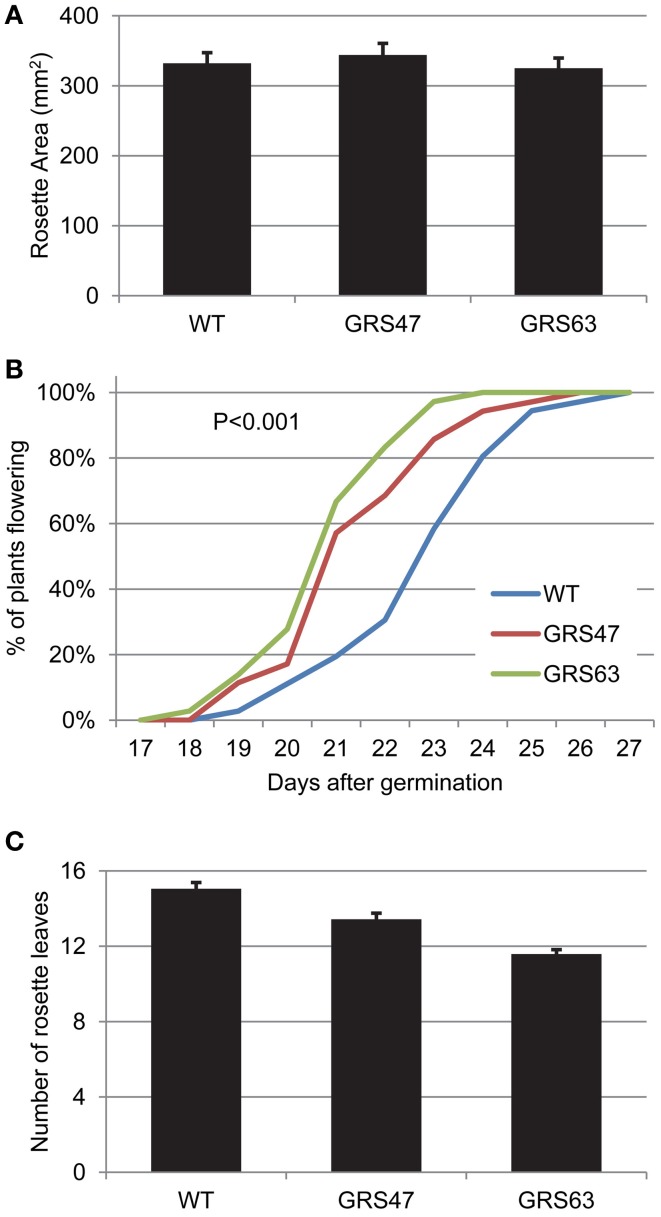
**Analysis of vegetative growth and the transition to reproductive growth in WT and transgenic lines. (A)** Rosette area at 17 dag; average and SE values from 12 replicates. **(B)** Percentage of plants flowering relative to days post germination. **(C)** Number of rosette leaves at the time of flowering. Average and SE, *n* = 36.

Leaf formation and rosette expansion was equivalent in WT, GRS47 and GRS63 at 17 dag (Figure 6). However, the transgenic plants did flower earlier than WT plants: 50% of GRS47 and GRS63 plants had visible inflorescences at 21 dag while WT plants reached the same threshold 23 dag (Figure 6). Furthermore, at flowering onset, GRS47 and GRS63 had fewer leaves than WT, arguing that the plants were not merely growing faster, but were transitioning to reproductive growth at an earlier developmental stage (Erickson and Michelini, 1957).

### Aphid behavioral assays

**Figure 7:**
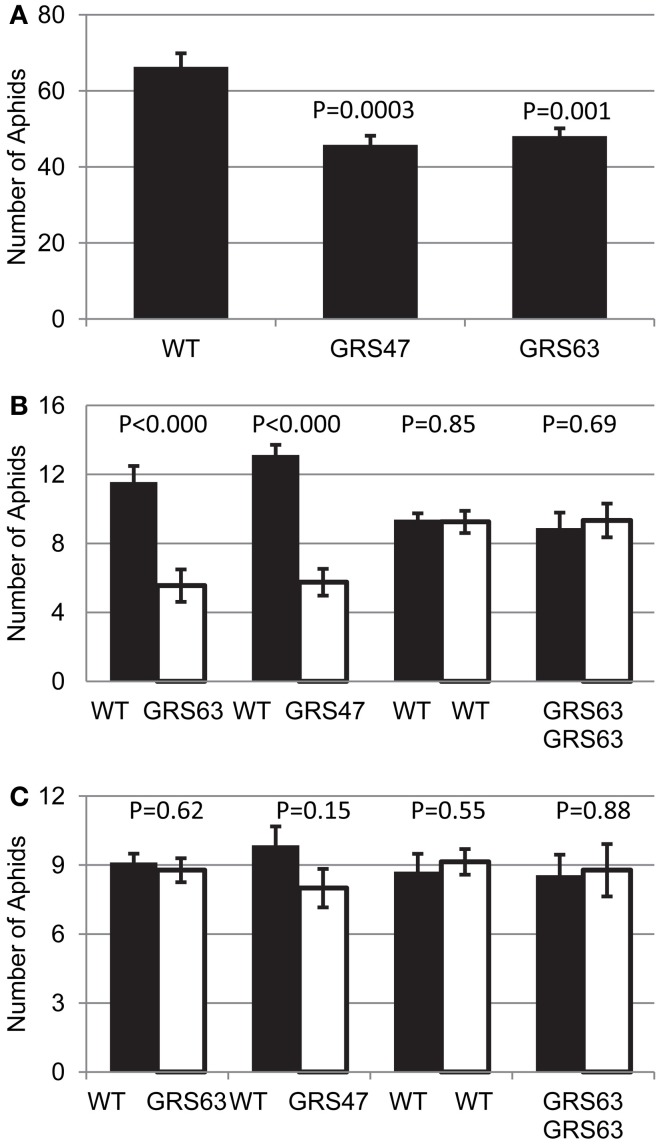
**Aphid behavior on WT and RFO-producing plants. (A)** Aphid populations on each plant after 48 h in a “no-choice” experiment (starting population = 20 adults). Average and SE, *n* = 12. **(B)** Adult aphids settled on each plant after 24 h in a “choice” experiment (starting population = 20 adults, released equidistant between the indicated plants). **(C)** As in **(B)**, but 8 h after aphid release. **(B,C)**, average and SE, *n* = 9. *t*-test *p*-values are indicated on each graph.

RFOs play a role in stress tolerance, including drought-, cold-, and oxidative-stress tolerance (Bailly et al., 2001; Taji et al., 2002; Nishizawa et al., 2008; Peters and Keller, 2009) and Gol may be a signaling component of induced systemic resistance caused by pathogens (Kim et al., 2008; Cho et al., 2010). Because of these roles in stress tolerance and the apparent presence of RFO in the translocation stream of GRS47 and GRS63, we tested for an impact on the behavior of a prevalent phloem-feeding insect, green peach aphid (*Myzus persicae* Sulzer*)*. To determine if transgenic plants producing RFOs influence aphid growth, growth on WT and transgenic plants was compared in “no-choice” bioassays, in which 20 mature individuals were placed directly on well-separated control or experimental plants, and aphids counted 48 h later. The aphid population was smaller on plants producing RFOs than the WT plants (Figure 7A). To determine if RFO accumulation impacts aphid settling on the transgenic plants, “choice” experiments were conducted in which 20 aphids were placed equidistant between a WT control and a transgenic plant growing in the same pot, and the number of adult aphids settling on each plant determined after 8 and 24 h. As controls for the choice bioassays, aphids were given a choice between two WT plants or two GRS63 plants growing in the same pot. Aphids had a strong preference for WT plants over RFO-producing plants after 24 h (Figure 7B), but importantly, there was no significant preference after 8 h (Figure 7C). Since green peach aphid begins feeding from sieve elements within 1–3 h of release on Arabidopsis (Pegadaraju et al., 2007; Louis et al., 2010), these results indicate that aphids did not show preference until after they began feeding on different plants and that there is no difference in the initial attraction to either plant. These results from choice and no-choice feeding experiments indicate that RFO-producing plants negatively impact aphid colonization.

**Figure 8:**
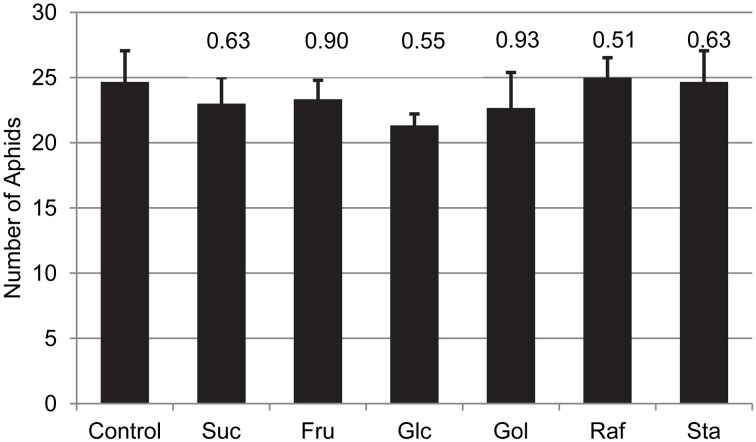
**The impact of the indicated sugar on aphid populations fed an artificial diet for 96 h**. Three adult aphids were released into the feeding chamber and allowed to feed on the diet through the sachet. The total number of aphids (adults + nymphs) were determined 96 h later. Average and SE, *n* = 3 replicates; *t*-test *p*-values, relative to diet without supplemental sugar are indicated on the graph.

To test if this resistance to the green peach aphid is a direct or indirect effect of the engineered sugars, aphid fecundity was measured on artificial media consisting of amino-acids, mineral salts, vitamins and 500 mM Suc (Mittler and Dadd, 1965), and supplemented with 50 mM of individual test sugars (Glc, Fru, Suc, Gol, Raf, of Sta). After 4 days, no differences in aphid populations were observed among treatments (Figure 8). These results indicate that the resistance to aphid colonization among the RFO-producing plants is not a direct toxic effect of the engineered sugars. The synthesis of RFOs may contribute to other defense responses that reduce the suitability of these plants as hosts for aphid pests.

## Discussion

In these studies, symplasmic phloem-loading biochemistry was engineer into the phloem of a plant that employs SUTs to load Suc from the apoplasm. The aims of these experiments were 2-fold: (1) to assess the efficiency of RFO synthesis in the phloem and (2) to gauge the efficiency with which RFO from the companion cells enters the translocation stream for long-distance transport. In addition, the growth and development of the engineered plants were monitored, and since the intent was to alter the contents of the translocation stream, the impact on the feeding behavior of green peach aphids (*Myzus persicae* Sulzer) was also tested.

Arabidopsis phloem loads from the apoplasm using a SUT encoded by *AtSUC2*. Metabolic engineering of the RFO pathway in the phloem, after SUT-mediated loading, was accomplished by introducing genes encoding GolS, RafS, and StaS driven by minor-vein and companion-cell specific promoters. Transgenic Arabidopsis lines GRS47 and GRS63 did not show alterations in rosette area at 18 dag but both flowered earlier than WT. It is possible that increasing sugar export in the transgenic plants caused earlier flowering since it is well-known that elevated sugar-levels, either endogenous to the plant or added exogenously, can accelerate the transition to flowering (Bernier, 1988; Corbesier et al., 1998). Manipulating *SUT* expression, and presumably sugar distribution, is shown to lead to earlier flowering (Sivitz et al., 2007; Chincinska et al., 2008), and similarly, manipulating the sugar content through metabolic engineering may impact flowering.

Despite the use of promoters specific to the companion cells of minor veins, RFO accumulated to greater than 50% of the total soluble carbohydrate without a compensating decrease in Glc, Fru, Suc, or starch. Although RFO accumulated to 50% of total soluble sugars (~2 nmol/mg fwt combined Gol, Raf, and Sta compared to ~3.5 nmol/mg fwt total soluble sugar in GRS47, 8 h into the illuminated period, Figure 1A), this is a relatively small amount of total, non-structural carbohydrate in leaves: Starch, as measured here, ranged from just below 10 nmol/mg fwt (Glc equivalents) at the end of the dark period to over 50 nmol/mg fwt 8 h into the light period, which is consistent with work by others (Blasing et al., 2005; Lunn et al., 2006). This normal cycling of carbohydrate pools is ~25-fold more than the RFO found in these lines.

Glc, Fru and starch showed increases between the dark and light period consistent with published results (Lunn et al., 2006). In WT plants, Suc also showed a modest (2-fold) increase between the dark and light period, consistent with published results (Blasing et al., 2005; Lunn et al., 2006), but changes were not observed in either transgenic line. It is intriguing that the transgenic lines exuded more Glc, Fru and Suc than WT (Figures 4, A1), but a lack of increase in rosette Suc cannot be directly linked to sugar increases in exudations with the experiments reported here, and the significance of neither phenomenon is clear. RFO levels in the transgenic plants did not vary from the end of the night period to 8 h into the light period, arguing they are not influenced directly by photosynthesis or by fluctuations in other carbohydrates.

Furthermore, photosynthetic radiolabeling with ^14^CO_2_ revealed that C flux through the engineered pathway is very low: The highest incorporation was only 2% of the total, whereas hexose sugars had as much as 30% of the total and Suc had as much as 80%. Suc is the first non-phosphorylated sugar of photosynthesis and high incorporation is expected, whereas much of the non-phosphorylated hexose is in the vacuole and removed from central metabolism. The low specific activity of the RFOs (i.e., low ^14^C incorporation during 20 min but high levels of unlabeled product) argues that RFO synthesis and turnover are low—lower than flux through the non-phosphorylated hexose pool—and that the high amounts result from gradual accumulation. Similar conclusions were drawn from transgenic tobacco expressing *CmGAS1* and producing low specific-activity Gol (Ayre et al., 2003b). The results presented here extend those findings and also argue that Raf and Sta in the transgenic plants are not created from stored Gol and recently photosynthesized Suc. These findings are also similar to those of Hannah and colleagues who showed poor Raf synthesis in the companion cells of potato (see below) (Hannah et al., 2006). However, this is in sharp contrast to plants that naturally transport RFO such as coleus (Turgeon and Gowan, 1992), cucurbits (Beebe and Turgeon, 1992), and catalpa (Turgeon and Medville, 2004), in which RFOs become quickly labeled to high specific activity, and in the case of catalpa, the galactose moiety becomes more strongly labeled faster than sucrose (Otto, 1968).

That RFOs in natural symplasmic loaders have high specific activity but that these engineered plants do not, and that these engineered plants do not have higher levels of RFO in the phloem, are puzzling. Apoplasmic loaders should have ample reduced carbon in the companion-cell cytoplasm, the engineered proteins are thought to localize to the cytoplasm (Keller and Pharr, 1996), and the plasmodesmata-pore units between companion cells and sieve elements are open to diffusion of 10 kDa dextrans (Kempers and van Bel, 1997; Knoblauch and van Bel, 1998) and 67 kDa proteins (Stadler et al., 2005). Based on these principles, there should not have been a limitation to RFO production or transport. Further work on the biochemistry occurring in the companion cells of apoplasmic loaders and the intermediary cells of symplasmic loaders is required to resolve why apoplasmic loaders do not effectively produce and transport RFOs. Hannah and colleagues came to a similar conclusion in their study (Hannah et al., 2006). The precursors for galactinol, UDP-Gal and myo-inositol, or flux through the pathways leading to these, may be insufficient for higher level production. These studies would be worthwhile since beyond the pursuit of basic science, there is substantial interest in engineering sugars and sugar derivatives for biotechnology (Patrick et al., 2013).

A probable cause of the high levels of RFO in both rosettes and roots, despite the low-specific activity, is diffusion away from both the source and sink phloem and gradual sequestration in inactive metabolite pools. In rosettes, estimates of minor-vein volume and leaf micro-dissection show that the RFOs were distributed throughout the leaves, and prior work with transgenic tobacco producing Gol in the minor veins showed similar distribution of Gol, including an equal spread between the apoplasm and cellular compartments (Ayre et al., 2003b). In roots, RFO accumulation equaled or exceeded that in rosettes, and likely had similar distribution away from the phloem. This, however, is not as surprising as the distribution in the leaves, since symplasmic solute distribution in sink organs is well-known (Lalonde et al., 2003).

How are the sugars moving from the sites of synthesis to be distributed through the entire leaf? The frequency of plasmodesmata connecting SECCCs to surrounding cells (phloem parenchyma or bundle sheath) has been used to represent how “open” the phloem is to symplasmic nutrient transport (Turgeon et al., 2001). Phloem anatomy was grouped by plasmodesmata abundance by Gamalei (1989, 1991) as abundant (Type 1), relatively abundant (Type 1-2a), and infrequent (Type 2a and Type 2b if they have transfer cell morphology). Surveys of this nature place Arabidopsis as Type 1-2a (relatively abundant plasmodesmata, 1–10 μm^−2^) (Haritatos et al., 2000b) and these relatively abundant plasmodesmata are strong candidates for allowing RFO movement from the veins to surrounding mesophyll.

Related to this, Hannah and colleagues produced Gol and Raf in potato companion cells using the *rolC* promoter (Hannah et al., 2006). Gol accumulated to 4.55 ± 0.56 nmol/mg fwt, but Raf only accumulated to 0.02 ± 0.00 nmol/mg fwt. When the *Cauliflower mosaic virus 35S* promoter was used for metabolic engineering, Raf accumulated to 35-fold greater levels, showing that there was not an intrinsic block to Raf accumulation in the leaves. Solanaceae species (e.g., potato) are classified as Type 2a (infrequent plasmodesmata; 0.1–1.0 μm^−2^) (Turgeon et al., 2001). Minor vein anatomy and plasmodesmata frequency therefore correlate with RFO accumulation in the source leaves of these engineered plants: relatively-open-Arabidopsis accumulates Gol, Raf, and to a lesser extent Sta, whereas relatively-closed tobacco and potato accumulate only Gol. Perhaps the open architecture of Arabidopsis allows Raf and Sta to diffuse more freely from the phloem to surrounding cells than the closed architecture of the Solanaceae. Gol movement may be by a different mechanism, such as broad specificity channels, as previously proposed (Ayre et al., 2003b). An important caveat to this is that plasmodesmata frequency as described by Gamalei does not measure porosity, and plasmodesmata are dynamic in their ability to open and close (Maule et al., 2011; Burch-Smith and Zambryski, 2012).

Although GRS47 and GRS63 had low levels of RFO in the phloem translocation stream, there was a strong impact on green peach aphid vitality and colonization. Aphids are phloem-feeding insects that constitute a biotic stress since they extract nutrients and reduce plant vigor. To test for an impact of RFO production on aphid feeding, “no choice” fecundity tests and “choice” feeding preferences were conducted. Transgenic Arabidopsis producing RFOs were a less preferred host for aphids and aphid reproduction was reduced. There were no significant preferences between WT and transgenic plants 8 h after aphid release, suggesting that the resistance to aphids is unlikely to result from chemicals released at the surface of plants. The preferences after 24 h could have resulted directly from the RFOs in the transgenic plants. It is worth noting that many animals cannot digest the α Gal1-6 linkage of RFOs, and RFOs can cause bloating, particularly in mammals (Martinez-Villaluenga et al., 2008). On the other hand, green peach aphid is a significant pest on cucurbit crops that translocate much higher levels of RFO than observed in the phloem sap of these transgenic Arabidopsis plants. Aphid feeding on synthetic media was used to test if RFOs directly impacted aphid behavior. Aphids did not show differences in reproduction 4 days after they were fed on synthetic media with or without RFOs and this indicates that the non-preference of aphids for the transgenic plants is not a result of a toxic effect of the RFO.

These results that RFO do not directly deter feeding, are consistent with those of Hewer et al. (2010) in which choice feeding behavior for several aphid species was tested on synthetic media against an extensive array of sugar concentrations, combinations, viscosities, and pH. In these studies, *M. persicae* preferred nutrient media with amino acids and Suc over a broad range (~10–25%, or 292–730 mM), and in the absence of Suc, Raf was the preferred tested sugar. With Suc and Raf equally combined (219 mM total), *M. persicae* preferred pure Suc (219 mM), but this was attributed to an attraction to high concentrations of Suc rather than a deterrence by Raf. High concentrations of Suc is an important cue for aphid probing and feeding behavior (Hewer et al., 2011). The deterrence observed on GRS47 and GRS63 relative to WT is therefore unlikely to be a direct effect of the exotic sugars, or the apparent differences in sugar concentration suggested by the exudation studies. Rather, the RFOs may act as signaling components of the systemic resistance protecting plants against aphids. Another possibility is that RFO accumulation in the transgenic plants may promote a general stress response which in turn results in compounds that deter aphids.

## Conflict of interest statement

The authors declare that the research was conducted in the absence of any commercial or financial relationships that could be construed as a potential conflict of interest.
